# Typhoid Fever

**Published:** 1911-10

**Authors:** Thomas Northen

**Affiliations:** Ashland, Alabama


### TYPHOID FEVER.

DR. THOMAS NORTHEN, ASHLAND, ALABAMA.

Typhoid Fever is a disease known in all civilized countries. In its malignant form there is, perhaps, no disease common in this country that is more fatal, excepting pneumonia. Owing to its extensive range of complications, the long continuance of an attack, its spread in families and communities and the great suffering of its victims, there is no disease more to be dreaded. Its presence in any community creates alarm, and no physician meets the disease without more or less anxiety. It was first considered as being identical with Typhus Fever and it is only about one hundred years since its pathology began to be understood. It was then that it was found to be a separate disease and since that time it has almost constantly engaged the thought and attention of the greatest medical scientists.

About three decades ago the microscopical researches of Eberth, Koch, Gaffy and others were rewarded by the discovery of a germ, the Bacillus Typhosus, which has been aemonstrated to be the cause of Typhoid Fever. Further researches discovered the habitat of the germ outside of the body and these discoveries laid the foundations for intelligent plans for prophylaxis and for the general management of the disease and brought about the antiseptic treatment which has shortened the course of the disease, moderated its violence, lessened the suffering of the patient, decreased the mortality rate and, to some extent, shorn the malady of its terrors.

The literature on typhoid fever is so extensive and so reputable that I could not be expected to bring before this intelligent body anything new on the subject. In a paper like this I could only be expected to touch on a few divisions, giving a few general ideas from the view-point of a country doctor, addressing these ideas mainly to the rural communities and to the doctors who serve them.

The pathology of the disease, while found in nearly every organ and tissue of the body, is located mainly in the intestinal canal, about the lower part of the ileum, involving the glands of Peyer, but it is frequently found above and below this point, giving rise to a catarrhal condition of the whole mucous membrane of the intestinal canal. These glands are found in a swollen condition and the nutrition is cut off, which results finally in strangulation of the glands, causing ulceration and sloughing. The germ Bacillus Typhosus is found in these glands, in the glands of the mesentery, in the spleen, in the bone marrow, in the liver and in the bile. It is found also in the blood and in the rose colored spots. They are discharged from the body in the urine, in the sweat and in the mucus of the mouth. After about the tenth day of the disease they are discharged from the bowels in large quantities, and the urine may contain the germ for months after recovery. These facts are full of suggestions in the way of successful prophylaxis.

Outside of the body the germ is found in polluted wells and springs, and these are the most fruitful source of infection.

The germ may be inhaled, but almost invariably it is swallowed in contaminated food and water. The infection may be carried to the mouth by the hands of those who handle patients sufferng from the disease. The disease is most frequent in thickly settled rural communities, in working camps, and towns and cities that have suddenly developed from small villages, and where sanitary knowledge has not kept pace with the increase of population. If communities would be free from the disease they should observe great cleanliness about their premises.

While filth will not cause the disease, it furnishes a nidus or home for the germ to live in and muliply, and, for the want of proper drainage, this filth, with the germ, is washed into wells and springs, and in localities where the earth is of a porous, gravelly nature, and where filth and water are allowed to stand in holes and ponds, the germ may filter, with the water, through the earth into the underground channels and be carried into wells. In order to prevent infection from these sources it is necessary to remove all uncultivated vegetation from the premises, to keep the ground swept of filth, to procure good drainage and see that water is not allowed to stand in holes. All filth should be burned, closets should have receptacles for excrement and these should be changed in from one to four weeks according to the amount of filth accumulated. Lime should be used freely each time the closet is used.

Wells should be looked after closely. Nothing should be found about a well except a bucket. Wash bowls, vegetables, fruit, and implements of every kind should be carefully kept away from the wells, and wash tubs and troughs for watering stock and washing clothing should never be allowed near a well. The handling of the well bucket by persons of filthy habits may infect the well.

About thirty years ago there was introduced into the country a well curb and a machine that would tilt and empty the bucket without handling it. The top was closed and filth could not possibly get in unless by intention. This was a good arrangement but it had one fatal defect. The platform upon which this machine rested was arranged so that persons had to stand on the platform to draw water. Where this platform was made of boards or plank the filth deposited by the feet would filter through the joints of the oor or be washed through into the well, causing the water to become filthy and sometimes causing infection.

With a platform covered with sheet iron and joined so as to be impenetrable and a perfectly closed top, this would be an ideal curb. Pumps are more easily protected than other machines for drawing water, and are to be preferred. For the platform, whether for pump or for machine above described, I would recommend a cement slab, inclined downward from the center with an opening in the middle sufficient for the pump or well bucket to pass.

To preevnt further infection in families where the disease has developed is a matter of greatest importance, and should receive the prompt attention of the medical attendant.

The family should be instructed as to the nature and sources of infection and the consequences of neglect.

As to the management of the patient: He should be placed in a comfortable room, well ventilated, but should be kept free from strong currents of air. This room should be away from noise and from the odor of food in preparation. Every article should be removed from the room except such as are necessary for the comfort and convenience of the patient and nurse.

A mattress is the best bed if not too uncomfortable for the patient. Covers should be light but sufficient to prevent any uncomfortable sense of chilliness. The room should be shaded to suit the patient, and at night should be without a light if the patient prefers. The light should never be allowed to shine in the face of the patient. In the winter season where the room is warmed by a fire-place the patient should be so place dthat he will not be affected by the heat of the fire.

The bed and body linen should be changed every day, the patient sponged all over with soap and water, the soiled linen placed at once in an antiseptic solution then boiled. Towels and napkins, together with all utensils should be sterilize,d and kept separate from those used by the family. The stools and urine should be disinfected thoroughly before being removed from the room, and the nurse should disinfect the hands after handling the patient or anything that has come in contact with the patient.

The danger of the house fly should not be overlooked. The doors and windows should be screened and flies kept entirely from the room. The discharges should be carried a good distance from the house and buried, burned or emptied in a place where they would be exposed to the direct rays of the sun for the destruction of any germs that may have escaped the disinfectant. The patient should be allowed cold water freely but I do not favor ice cold water. The water may be acidulated with lemon juice but I do not favor the use of lemonade. He should be free from business cares, and news that would excite much interest as well as newspapers shoudl be excluded. Children should not be allowed their toys. I have known the use of chewing gum to cause a rise of temperature and pulse of children. A thermometer should be kept by the nurse and the temperature and pulse should be recorded twice a day, but frequent use of the thermometer should be prohibited. In the early stage of the disease food should consist of light broths, given at the usual meal time.

Later, when the patient begins to show emaciation and loss of strength, the diet should afford more nutriment, and if the patient loaths his food, the flavor of vegetables such as the patient has a relish for may be added, such as the milk of green corn, tomatoes, etc., and when nutriment is very important to sustain the strength of the patient, fruit juices may be used, such as grape juice, cider, pickles of various kinds, and the juices of meats that may be relished. Of course it is only the juice of the pickles etc., that may be used.

Of all the articles of diet I have tried, buttermilk has been the most acceptable to the patient as well as the least harmful, and the relish for it lasts longer. It should be served cold and fresh and free from the presence of butyric acid.

I have found it sufficient in nutriment for all stages of the disease, but it should be alternated with the broths or a little bread in which there is salt enough to supply the needs of the system.

In cases where there are no complications, few medicinal agents are necessary. I usually begin the treatment with calomel. If the tongue is red and raw, and the bowels loose, there is a probability of the disease having been in progress for several days, I omit the calomel. For the first four or five days, if the bowels are inclined to constipation I repeat the calomel again. After the first week, if there seems to be a tendency to constipation I substitute powdered blue mass for the calomel, and if it seems indicated I use small doses of powdered blue mass during the second week. The bowels should act from one to three times a day. Salol and bismuth are used from the beginning to the end of the fever at three hour intervals, but the patient is never waked to give medicine. I use a diuretic in connection with the above, either spirits of nitre or bi-carbonate of potash. Quinine is given from the beginning for the first week in the usual dose for malarial fever, especially if there are indications of malaria in the case. For the diarrhoea that sometimes ushers in the case and for that which sometimes develops later on, I use tanigen and tincture opium, or opium and nitrate of silver, both of which I have used successfully, but I think opium and nitrate of silver the better remedy.

For the bilious vomiting in the early stages of the disease which so tries the patient and the doctor, I have found purgative doces of calomel almost invariably successful.

In athenic form of the fever when there are redness of the conjunctivae, a full pulse, photophobia, headache, insomnia and active delirium, I have found veratrum a most successful remedy, and it should be continued in the case till those active symptoms have completely subsided, even till the disease has spent its force.

To completely overcome the active delirium it is sometimes necessary to combine morphine with the veratrum. In an epidemic that prevailed in Clay County about twenty-eight years ago, the addition of veratrum to the other treatment seemed to lessen the mortality very perceptibly, and to add greatly to the comfort of the patient. As to hydrotherapy, I have never used it to the full extent. I believe in it, and hospital reports of its success are highly encouraging, but I do not think it can be carried out in all its details in country practice. From the beginning of the disease or when the temperature is over 100, I use a water bottle under the head and cold cloths to the head constantly, if comfortable to the patient. Before water bottles were known to the country doctor, I used a constant douche to the head till it felt cool and as soon as the heat began to manifest itself again, the dauche was renewed. The arrangement for this douche consisted of a bucket of water above the head, and a vessel below to receive the water, and a rubber tube in the form of a siphon to conduct the water to the head of the patient. This application afforded so much comfort that the patient would sometimes sleep for hours while the douche was being applied. When the temperature is very high I use cold sponging to the body, but bathing the extremities is of doubtful utility, especially when they remain cool after the bath. The douche would lower the temperature from one to two degrees and relieve the nervous erethysm of the patient which seems to be the greatest evil of high temperatures. In children, however, when the fever is allowed to run high and in cases of sudden onset, the cold to the head is likely to shock the nervous system and aggrovate the conditions which it is intended to relieve, especially when applied suddenly. In these cases I have found acetanilid a valuable remedy. It lowers the temperature, quiets the nerves and allows time to bring to bear the cold application to the head.

In regard to the use of the coal tar derivatives, I use them frequently in the beginning, principally as analgesics, to overcome hyperaesthesia of the nervous system, to relieve pain, but as soon as the alimentary canal ahs been emptied and disinfected I discontinue them and use opiates if anodyne treatment is called for.

While I consider them as especially useful in the ephemeral fevers of childhood, I regard them as worse than useless in the continued forms of fever. I will add that according to my experience in fevers, high temperatures are not of so much consequence as authors generally state. I am never concerned but little over a temperature of 104 or even 105 if pulse is under 100, and an evening temperature of 103 or 104 preceeded by a morning temperature of 100 to 102, after the second week, need not excite any apprehension.

I have found the pulse by far the most reliable index to the condition of the patient, and it will always announce the approach of danger. In regard to the use of alcoholic stimulants, I have found but few cases in which they were beneficial. As heart tonics or stimulants they are worthless. As a food I have never observed any benefits from their use. It is true that fluid extract of malt is a fine restorative, but I do not think that the benefit results from the alcohol. It is more probably the effect of the malt. I believe that there are a few cases in which alcohol would be beneficial, and they are those in which there is not a sufficient supply of blood in the brain as is manifested by the whiteness of the conjunctiva, low muttering delirium, and a tremor of the tongue and limbs with inability to speak. I have a case in mind. I wall called to see a man about twenty-five years old, who had been under treatment for typhoid fever about three weeks. He was very nervous and feeble and on account of the tremor of the tongue he could not utter a word, though he seemed anxious to do so. His pulse was about 150 and his temperature 104. His tongue was red and dry and very tremulous, and his eyes were white.

There were three doctors in consulation and after carefully considering the case, we all agreed that it was impossible for the patient to recover, so we departed to our respective fields of labor without having left any advice or treatment. The next news we had from the case he had fully recovered, and upon inquiry we found that after we abandoned the case a neighbor came with a bottle of whiskey and took charge of the case and under the effects of whiskey alone the patien recovered. During the consulation one of the doctors remarked that the whiteness of the patient's eyes indicated deficiency of blood in the brain and that alcohol might change this condition. Considering the case hopeless the remedy was not tried, and this remedy in the hands of this neighbor accomplished the cure.

Whiskey, we all know, has an elective or special action on the blood vessels of the brain. It, no doubt, increases the quantity of blood in the brain by paralyzing the vasso-motor nerves and there are exceptional cases, no doubt, in which it is highly valuable.

*Discussion on the paper of Dr. Northen, Typhoid Fever.*

Dr Langley,— I do not think I can add anything to this valuable paper. Do not know that my experience is as great as some of the older doctors. Think one great difficulty is, to distinguish between typhoid and malaria fever. I will leave the subject to the older practictioners to discuss.

Dr. Baker,— I would like to ask the Doctor to kindly give his opinion of alcohol in the routine treatment of typhoid fever. I do not think alcohol should be used in the routine treatment, unless as a vaso-motor stimulant, when it is sometimes effective. Alcohol is a narcotic, or degressant, and its value as a food is questioned. I believe the profession is using it less and less each year.

Dr. Gautier,— I heard the valuable paper, and thank the doctor for it, as it is a subject in which every doctor is interested. I do not use alcohol in the routine treatment, use alcohol in water for sponges. I am an advocate for the old treatment of turpentine. As a food I use peptonoids a great deal, and as a stimulant I use strychnine.

Dr. Northen in closing.— I thank the doctors for their discussion of my paper, have nothing further to say in closing except to answer Dr. Baker. In my early practice I used alcohol, but never found it of much value, so of late years I do not use it, but some recommend it highly.

